# Tackling Barriers To Increase Early Palliative Care Access For Older Adults

**DOI:** 10.1093/geroni/igaf122.4017

**Published:** 2025-12-31

**Authors:** Natalie Martinez, Vanessa Gonzalez, Gabriela Zaragoza, Melida Juarez-Briggs, Anjuli Vasquez, Michael Mader, Sandra Sanchez-Reilly, Jennifer Barker

**Affiliations:** South Texas Veterans Health Care System, San Antonio, Texas, United States; South Texas Veterans Health Care System, San Antonio, Texas, United States; South Texas Health Care System, San Antonio, Texas, United States; South Texas Veterans Health Care System, San Antonio, Texas, United States; South Texas Veterans Health Care System, San Antonio, Texas, United States; South Texas Veterans Health Care System, San Antonio, Texas, United States; University of Texas Health San Antonio, San Antonio, Texas, United States; University of Texas HSC at San Antonio, San Antonio, Texas, United States

## Abstract

**Background:**

Early palliative care (EPC) has been shown to provide a myriad of benefits including improved quality of life for older adults. Despite this, there is continued underutilization of palliative care (PC) in the geriatric population, and many barriers to obtaining timely consultations.

**Methods:**

This Quality Improvement (QI) project utilized the IPAL-ICU screening tool with modifications (PCST) to prioritize PC consultations for seriously ill older adults. Building upon our original project “Increasing Palliative Care in the Progressive Care Unit”1, we expanded use of the PCST tool to two additional units within our veteran’s hospital from August 2023 -July 2024. Multiple QI strategies were used: dedicated RN-led QI project, RN unit champions, physician education campaigns, flyers, brochures, feedback gathering, and regular audits of progress.

**Results:**

N = 621 PCST screens completed. 494 unique veterans, 96% male, 398 new and 223 established. The median age was 73 with an interquartile range (IQR) of 64 to 78. Of the new patients, 122 were seen inpatient and 146 were transferred to outpatient consultations. The PCST contributed to an increase in 40% of inpatient consultations and 30% increase in outpatient consultations, allowing for earlier PC access for seriously ill older adults.

**Conclusion:**

PCST is an effective means of prioritizing PC consultations for seriously ill older adults. Implications for Research, Policy, or Practice: Our QI process has led to optimizing a standardization tool for screening which can be disseminated on a national level to successfully move PC consultations earlier for seriously ill older adults.

